# Spatial distribution of insecticide resistant populations of *Aedes aegypti* and *Ae. albopictus* and first detection of V410L mutation in *Ae. aegypti* from Cameroon

**DOI:** 10.1186/s40249-022-01013-8

**Published:** 2022-08-17

**Authors:** Matthew Montgomery, James F. Harwood, Aurelie P. Yougang, Theodel A. Wilson-Bahun, Armel N. Tedjou, Christophe Rostand Keumeni, Auston Marm Kilpatrick, Charles S. Wondji, Basile Kamgang

**Affiliations:** 1 U.S. Naval Medical Research Unit No. 3, Naval Air Station Sigonella, Italy; 2 Centre for Research in Infectious Diseases, P.O. Box 13591, Yaoundé, Cameroon; 3 Parasitology and Ecology Laboratory, Department of Animal Biology and Physiology, Faculty of Science, University of Yaoundé 1, P.O. Box 812, Yaoundé, Cameroon; 4 Laboratory of Vertebrate and Invertebrate Bioecology, Faculty of Science and Technology, Marien-Ngouabi University, Brazzaville, Congo; 5 Ecology and Evolutionary Biology Department, University of California, Santa Cruz, USA; 6 Vector Biology Department, Liverpool School of Tropical Medicine, London, UK

**Keywords:** Arbovirus, *Aedes aegypti*, *Aedes albopictus*, Insecticide resistance, Knock down resistance, Cytochrome P450 monooxygenases, Cameroon

## Abstract

**Background:**

Dengue (DENV), chikungunya (CHIKV) and Zika virus (ZIKV), are mosquito-borne viruses of medical importance in most tropical and subtropical regions. Vector control, primarily through insecticides, remains the primary method to prevent their transmission. Here, we evaluated insecticide resistance profiles and identified important underlying resistance mechanisms in populations of *Aedes aegypti* and *Ae. albopictus* from six different regions in Cameroon to pesticides commonly used during military and civilian public health vector control operations.

**Methods:**

*Aedes* mosquitoes were sampled as larvae or pupae between August 2020 and July 2021 in six locations across Cameroon and reared until the next generation, G1. *Ae. aegypti* and *Ae. albopictus* adults from G1 were tested following World Health Organization (WHO) recommendations and *Ae. aegypti* G0 adults screened with real time melting curve qPCR analyses to genotype the F1534C, V1016I and V410L *Aedes kdr* mutations. Piperonyl butoxide (PBO) assays and real time qPCR were carried out from some cytochrome p450 genes known to be involved in metabolic resistance. Statistical analyses were performed using Chi-square test and generalized linear models*.*

**Results:**

Loss of susceptibility was observed to all insecticides tested. Mortality rates from tests with 0.25% permethrin varied from 24.27 to 85.89% in *Ae. aegypti* and from 17.35% to 68.08% in *Ae. albopictus*. Mortality rates for 0.03% deltamethrin were between 23.30% and 88.20% in *Ae. aegypti* and between 69.47 and 84.11% in *Ae. albopictus*. We found a moderate level of resistance against bendiocarb, with mortality rates ranging from 69.31% to 90.26% in *Ae. aegypti* and from 86.75 to 98.95% in *Ae. albopictus*. With PBO pre-exposure, we found partial or fully restored susceptibility to pyrethroids and bendiocarb. The genes *Cyp9M6F88/87* and *Cyp9J10* were overexpressed in *Ae. aegypti* populations from Douala sites resistant to permethrin and deltamethrin. *Cyp6P12* was highly expressed in alphacypermethrin and permethrin resistant *Ae. albopictus* samples. F1534C and V1016I mutations were detected in *A. aegypti* mosquitoes and for the first time V410L was reported in Cameroon.

**Conclusions:**

This study revealed that *Ae. aegypti* and *Ae. albopictus* are resistant to multiple insecticide classes with multiple resistance mechanisms implicated. These findings could guide insecticide use to control arbovirus vectors in Cameroon.

**Supplementary Information:**

The online version contains supplementary material available at 10.1186/s40249-022-01013-8.

## Background

*Aedes-*borne viral diseases such as dengue, chikungunya and Zika are increasingly reported in different regions across the world, including Africa [1]. For dengue, approximately half the world’s population is at risk with an estimated 100–400 million new infections reported each year [1]. Vector control remains the cornerstone to prevent and fight against transmission. Use of insecticide based-control strategies is a common approach in response to arboviral disease outbreaks in order to quickly lower vector density and ongoing transmission [2], and is often the primary strategy to mitigate the threat of dengue, chikungunya, and Zika during military operations [3]. However, the increased use of insecticides may result in the selection of mosquitoes that carry genetic traits associated with insecticide resistance. Indeed, resistance to multiple insecticides used in public health has been documented in *Aedes aegypti* and *Ae. albopictus* in different locations across the world [4].

Insecticide resistance is usually achieved through two mechanisms: increased detoxification (metabolic resistance) and target insensitivity (target site resistance). Metabolic resistance through upregulation of detoxification genes is a common resistance mechanism in *Ae. albopictus* and *Ae. aegypti*, caused primarily by three main enzyme families, the monooxygenases (cytochrome P450s), glutathione S-transferases (GSTs) and carboxylesterases (COEs) [5, 6]. Target site resistance is caused by mutations in target genes such as acetylcholinesterase (*Ace-1*), the *GABA* receptor, and the voltage-gated sodium channel (*VGSC*) causing knockdown resistance (*kdr*) [5, 6].

Knockdown resistance is one of the main target site adaptations for both pyrethroids and dichlorodiphenyltrichloroethane (DDT). In *Ae. albopictus*, the *kdr* mutation is less prevalent with only four voltage-gated sodium channel (VGSC) mutations detected affecting two codons (1532 and 1534). In *Ae*. *aegypti*, many *kdr* mutations have been reported [4, 7, 8]. Among these mutations, the V1016I, F1534C and V410L mutations have been extensively investigated in pyrethroid-resistant *Ae*. *aegypti* populations from Asia, South America and, to a lesser extent, Africa. In Cameroon F1534C and V1016I mutations have been previously reported in *Ae. aegypti* [9, 10]. V410L is a novel *kdr* mutation, located in domain I of segment 6 of the VGSC. It was described for the first time in a pyrethroid-resistant *Ae*. *aegypti* laboratory strain originating from Rio de Janeiro, Brazil [7] and several years later in Africa [11]. Considering that this mutation has shown a potential to reduce the sensitivity of sodium channels to type I and II pyrethroids and to increase resistance when associated with F1534C [12], it is imperative to determine the current distribution of this mutation in natural *Ae. aegypti* populations in Cameroon. This is due to the fact that its presence can greatly impair the usefulness of a wide variety of pyrethroid insecticides, which currently constitute the major class of insecticides used in *Aedes* control [13, 14]. In addition, several cytochrome P450 genes have been found overexpressed in *Aedes* spp. populations tested in the field and demonstrating pyrethroid resistance, with evidence indicating a role in pyrethroids metabolism by *CYP9J28*, *CYP9J10*, *CYP9J26*, *Cyp9M6F88/87* and *CYP6P12* [4, 15–19].

Our goal was to quantify the insecticide resistance profile and mechanisms involved in the resistance of populations of *Ae. aegypti* and *Ae. albopictus* from six cities that span most of the 1000 km north–south extent of Cameroon. In Cameroon, *Ae*. *aegypti* is present across the country, while the distribution of *Ae*. *albopictus* is limited to the south, under 6°N [20, 21]. We selected insecticides commonly used for public health and military force health protection applications to mitigate disease threats.

## Methods

### Mosquito sampling and rearing

**Fig. 1:**
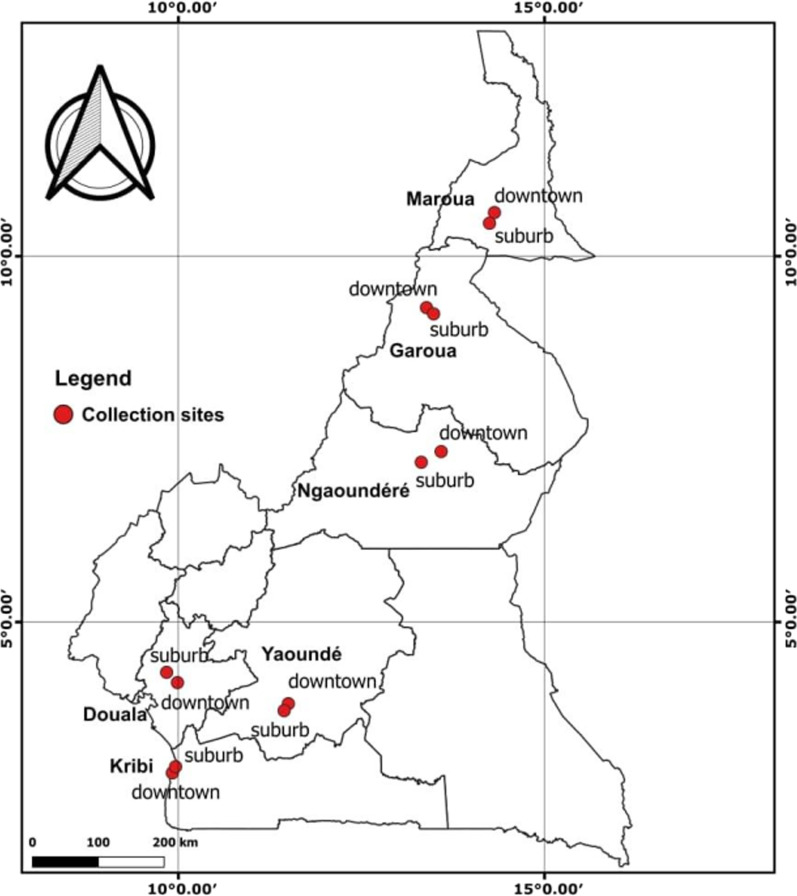
Map of study sites in six cities in Cameroon

*Aedes* mosquitoes were sampled as larvae or pupae between August 2020 and July 2021 in six locations across Cameroon: Douala (N 04° 02′ 729″, E 09° 42′ 142″), Maroua N 10° 37′ 284″, E 14° 18′ 381″), Garoua (N 09° 17′ 7776″, E 13° 23′ 288″), Ngaoundéré N 07° 35′ 414″, E 13° 57′ 365″, Yaoundé (N 03° 51′ 993″, E 011° 27′ 688″), and Kribi (N 02° 56′ 862″, E 09° 54′ 003″) (Fig. 1). Immature stages (field generation, G0) were collected from different potential larval habitats: domestic (e.g., jars, tanks), peri-domestic (e.g., used tires, discarded tanks), and natural (e.g., tree holes). In each location, larvae, or pupae from 20 positive larval habitats were collected, stored in plastic boxes, and transferred to insectary then pooled according to the location and land use category (urban and suburban) and reared to adult stage for identification using taxonomic keys [22, 23]. Mosquitoes identified as *Ae. albopictus* or *Ae. aegypti* were blood-fed and allowed to reproduce to generation G1 for adult bioassays. Mosquito populations were maintained at insectary conditions (temperature 27 ± 2 °C; relative humidity 80% ± 10%), and females were blood fed using hemotek membranes to complete their gonotrophic cycle. Two susceptible lab strains were used for this study: Vector Control Research Unit (VCRU) strain for *Ae. albopictus* and Benin lab stain for *Ae. aegypti*.

### Insecticide resistance bioassays

Bioassays were carried out according to the World Health Organization (WHO) protocol using 3–5 days old G1 generation mosquitoes. Four replicates of 20–25 females per tube were exposed to 0.03% and 0.05% deltamethrin, 0.05% alphacypermethrin, 0.1% bendiocarb, and 0.25% or 0.75% permethrin for 1 h. Mortality was recorded 24 h later; mosquitoes, alive or dead, after exposure were stored in RNA later or silica gel, respectively. Mortality rates were interpreted according to WHO recommendations: mortality rate of 98% or above indicates susceptibility, mortality rate between 90 and 97% suggests a possibility for resistance that should be confirmed with further bioassays, and mortality rate less than 90% indicates confirmed resistance [14].

### Adult synergist assay with PBO

To evaluate the effectiveness of insecticide detoxification by the mosquitoes, the specific enzyme inhibitor 4% piperonyl butoxide (PBO) was evaluated to address the potential role of P450s in insecticide resistance. Female adults, 3–5 days old, were pre-exposed for one hour to PBO-impregnated papers and then immediately exposed to the selected insecticide. Mortality was scored 24 h later and compared to the results obtained with each insecticide without synergist according to the WHO standards [14].

### Knockdown resistance (*kdr*) genotyping

Approximately 30 mosquitoes per city were genotyped for three different *kdr* mutations: V1016I, V410L and F1534C. Genomic DNA of 30 individual mosquitoes per population was extracted using the Livak protocol [24]. Three kdr mutations (F1534C, V1016I and V410L) were chosen for this study because these mutations have been described as involved in the pyrethroids resistance of *Ae. aegypti* mosquito [4, 25]. These mutations have been also reported in Africa [11, 15, 26]. Based on Moyes et al., review, F1534C and V410L are associated to insecticide resistance and V1016I is associated to insecticide resistance when combined to other *kdr* mutations. Genotyping of the V1016I, V410L and F1534C mutations was performed by real-time melting curve quantitative PCR [27]. Each PCR reaction was performed in a 21.5 μl volume PCR tube containing 2 μl of DNA sample, 10 μl of SYBR® Green (SuperMix), and 1.25 μl of each primer. The thermocycle parameters were: 95 °C for 3 min, followed by 40 cycles of (95 °C for 20 s, 60 °C for 1 min and 72 °C for 30 s) and then a final step of 72 °C for 5 min, 95 °C for 1 min, 55 °C for 30 s and 95 °C for 30 s.

## Gene expression

### RNA extraction and cDNA synthesis

For this experiment three groups of mosquitoes were used: unexposed (control) to insecticide individuals from G1, exposed G1 individuals that survived resistance assays (resistant), and susceptible (laboratory susceptible strains). For each group three replicates of 10 mosquitoes per species were performed. RNA was extracted using the PicoPure RNA Isolation Kit (Arcturus® Picopure RNA Extraction Kit Life Technologies, California, USA), following the manufacturer's recommendations. Quality and quantity of RNA obtained were assessed using a "NanoDrop Lite" spectrophotometer (Thermo Scientific Inc., Wilmington, USA) and stored at − 80 °C.

Extracted RNA was used to synthesize complementary DNA (cDNA) using the Superscript III kit (Invitrogen, Carlsbad, CA, USA) according to the manufacturer's instructions. The resulting cDNA was purified using a QIAquick spin column (QIAuick PCR Purification Kit, Qiagen) and diluted 2-fold to accommodate the volumes of the reaction.

### Quantitative-reverse transcriptase PCR

Candidate genes for expression analysis were chosen based on previous implications of involvement in metabolic resistance [4, 17]. Standard curve analyses were performed for each primer pair to check the specificity and efficiency of amplifications. Four cytochrome P450 candidate genes were chosen for analysis in *A. aegypti* (*Cyp9M6F88/87*, *Cyp9J28a*, *Cyp9J10,* and *Cyp9J32*) and only one in *A. albopictus* (*Cyp6P12*). The reactions were performed in a volume of 20 μl with 10 μl sybrGreen (Applied Biosystems, Texas, USA), 0.6 μl of each primer (10 μm), 7.8 μl of ddH2O, and 1 μl of CDNA, under the following conditions: 95 °C for 3 min, followed by 40 cycles of 95 °C for 10 s and 60 °C for 10 s. The relative expression level and fold change (FC) of each candidate gene compared to susceptible strains were calculated using the 2-ΔΔCT method, integrating the efficiency of the PCR [17] after normalization with housekeeping genes: Aaeg60sL8, RPF7, RSP7, and tubulin. All primer sequences and their origins are shown for *A. albopictus* and *A. aegypti* in Tables 1 and 2, respectively. The Mx Pro software integrated into the Agilent brand TaqMan machine was used (MxPro-Mx3005P v4.10, Stratagene, California, USA).

**Table 1:** Primer sequences for the evaluation of the level of expression of metabolic resistance genes by RT-qPCR and their origins in *Aedes albopictus*

Genes	Forward primer	Reverse primer	References
*Cyp6P12*	CGTGCGCTTTTGGGATTGAG	ATCGTCCGTGCCAAATCCTT	[17]
*RSP7*	AAGGTCGACACCTTCACGTC	CGCGCGCTCACTTATTAGAT	[17]
q*Tubulin*	CCGCACTCGAGAAGGATTAC	GTGGTTCGGTTTGACTTCGT	[17]

**Table 2:** Primer sequences for the evaluation of the level of expression of metabolic resistance genes by RT-qPCR and their origins in *Aedes aegypti*

Genes	Forward primer	Reverse primer	References
*Cyp9J10*	ATCGGTGTTGGTGAAAGTTCTGT	CATGTCGTTGCGCATTATCCC	[46]
*Cyp9J28*	CCACTGACGTACGATGCGA	GCCGATCAGTGGACGGAGC	[17]
*Cyp9M6*	TCGGTGCACAATCCAAACAAC	GTCGGGTACGACCAACGAAA	[18]
*Cyp9J32*	CGGTCCGCTTATGACGAAGAG	TTTGTTCGCTCCGAAGAGTGG	[47]
*RPS3*	AGCGTGCCAAGTCGATGAA	GTGGCCGTGTCGACGTACT	[18]
*Ae60sL8*	CTGAAGGGAACCGTCAAGCAA	TCGGCGGCAATGAACAACT	[17]

### Data analysis

Comparisons of mortality rates between species and land use category were conducted using generalized linear models (GLM) with a binomial distribution and logit link function using the *lme4* package in R version 3.6.1 (R: A language and environment for statistical computing. R Foundation for Statistical Computing, Vienna, Austria). The comparison of mortality rate after pre-exposure of mosquitoes to synergist and without pre-exposure to synergist was performed using a Chi-square test. The relative expression level and fold change (FC) of each target gene in field samples relative to the susceptible Benin (*Ae. aegypti*) or VCRU (*Ae. albopictus*) were calculated according to the 2^−^ΔΔ^CT^ method incorporating the PCR efficiency after normalization with the housekeeping genes. This analysis was performed using GraphPad Prism 8.0.2 (GraphPad Software, San Diego, California, USA). *P-*value < 0.05 was considered as statistically different.

## Results

### Insecticide resistance profile for *Aedes aegypti*

**Fig. 2:**
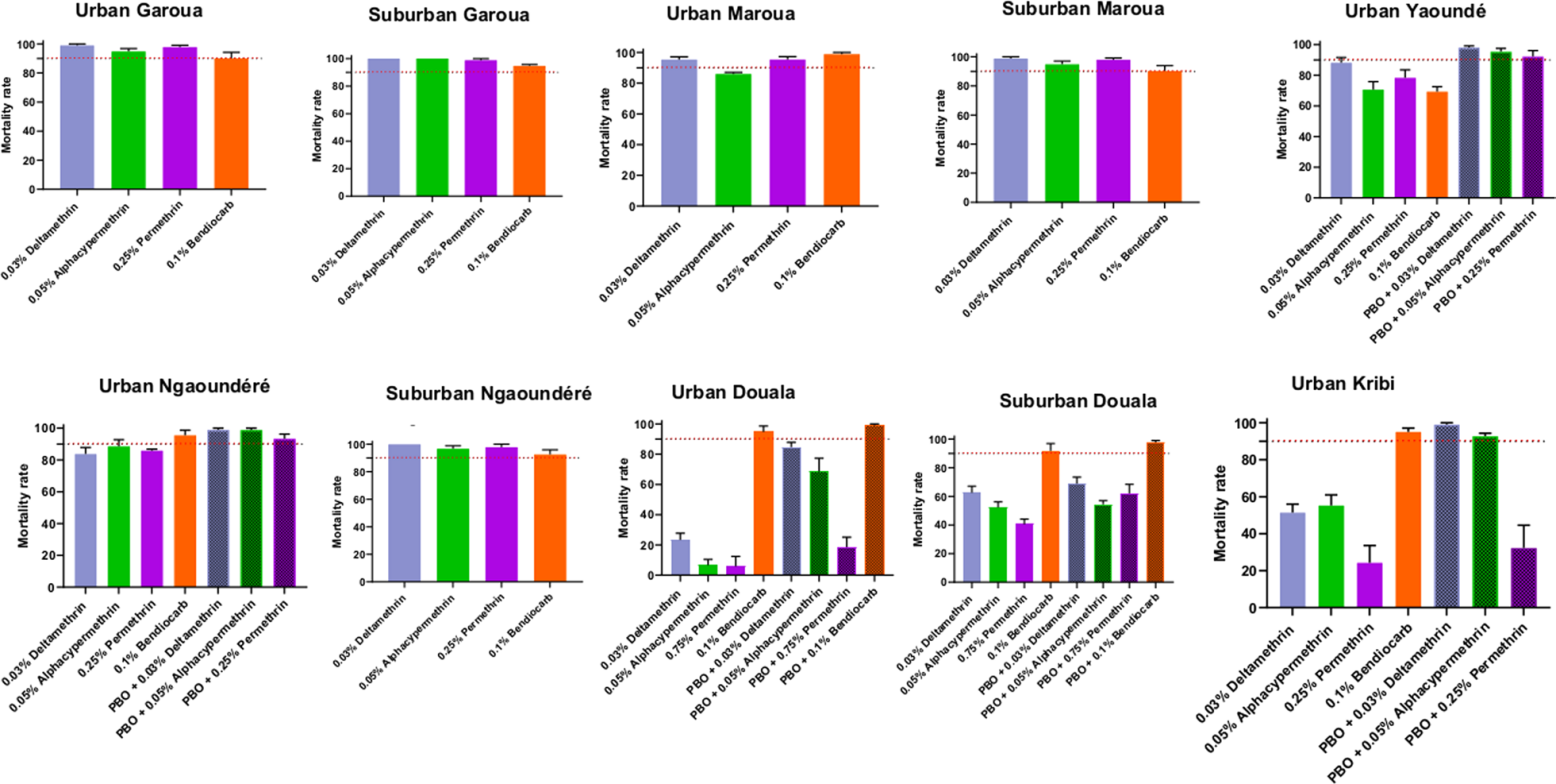
Mortality rates of adult *Aedes aegypti* from urban and suburban habitats in six cities in Cameroon 24 h after exposure to insecticides alone or with 1 h preexposure to synergist. Error bars represent standard error of the mean. PBO*:* Piperonyl butoxide

In total we tested 10 populations of *Ae. aegypti* with four insecticides (Fig. 2). Susceptibility for three pyrethroid varied among locations. To 0.25% permethrin, high resistance was observed with mortality rates ranging from 24.27% in urban Kribi to 85.89% in urban Ngaoundéré, probable resistance in urban Maroua (mortality rate of 95.43%); susceptibility in suburban Ngaoundéré, urban Garoua, suburban Garoua, and suburban Maroua (mortality rate from 97.82 to 98.80%). Remarkably, even when using 0.75% permethrin, which is triple the dose recommended for *Aedes* control*,* high levels of resistance were still observed. For example, the mortality rates to the 0.75% permethrin were 5.83% in urban Douala, and 40.48% in suburban Douala. For 0.03% deltamethrin, resistance was found in urban Yaoundé, urban Kribi, urban Ngaoundéré, urban and suburban Douala (mortality rates varying from 23.30% in urban Douala to 88.20% in urban Yaoundé); probable resistance in urban Maroua and susceptibility in suburban Ngaoundéré, urban Garoua, suburban Garoua, and suburban Maroua (mortality rate varying from 98.80% in suburban Maroua to 100% in suburban Ngaoundéré). Six of 10 populations tested were resistant to alphacypermethrin (urban Yaoundé, urban Kribi, urban Ngaoundéré, urban Maroua, and urban and suburban Douala); three others exhibited probable resistant (suburban Ngaoundéré, urban Garoua, and suburban Maroua) and the last one was fully susceptible (suburban Garoua). A moderate level of resistance was reported against bendiocarb with mortality rates varying from 69.31% in urban Yaoundé to 90.26% in suburban Maroua. In addition, probable resistance was detected in six populations with mortality rates ranging from 91.79% in Douala suburban to 95.50% in Ngaoundéré urban. Only the population from urban Maroua was susceptible to bendiocarb (mortality rate of 98.91%).

**Fig. 3:**
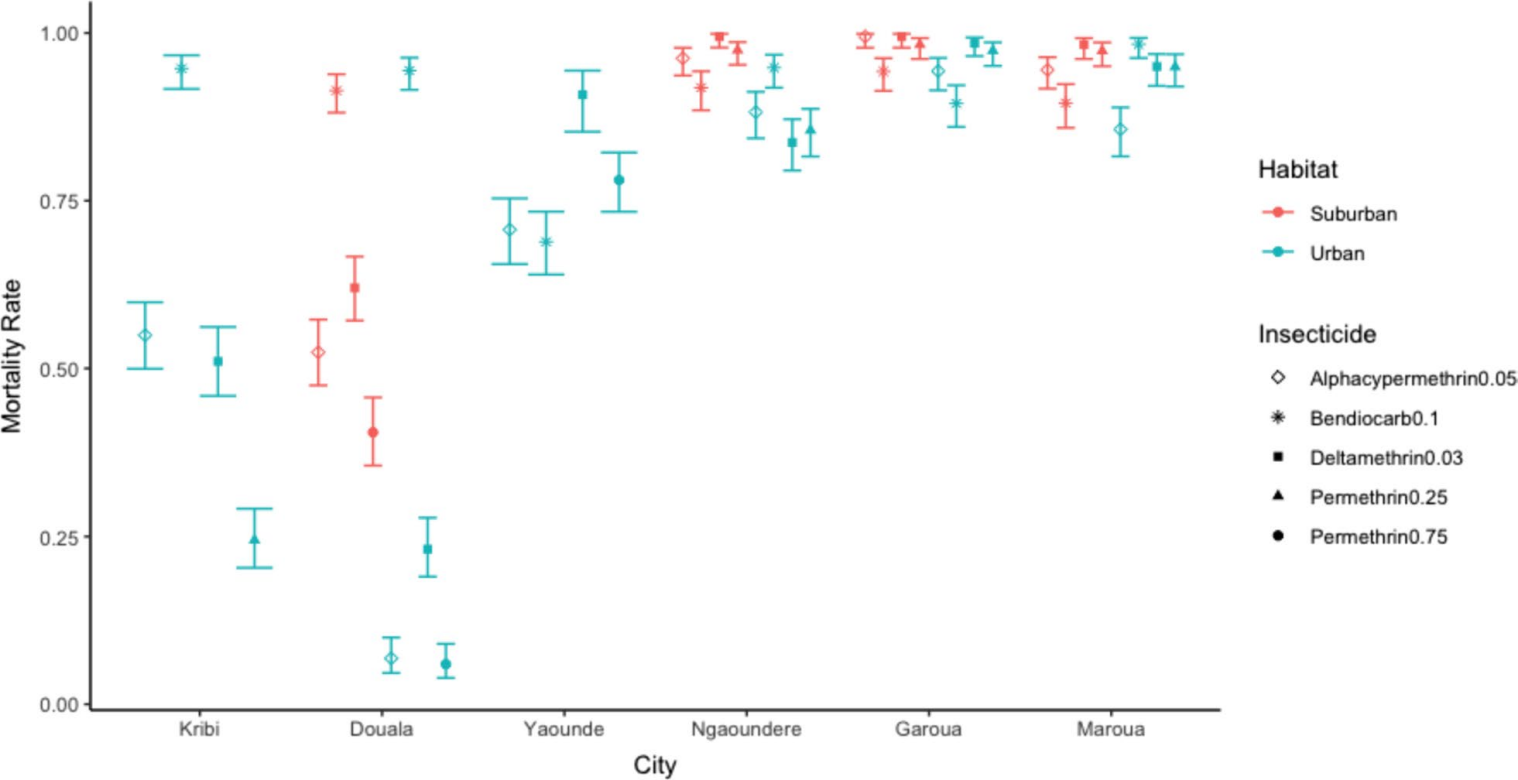
*Aedes aegypti* insecticide mortality (± 1 SE) between study cities and habitats. Cities on the x-axis are arranged along a northward gradient

The best fitting model for predicting insecticide mortality in *Ae. aegypti* was a triple interaction of environment (urban or suburban), city, and insecticide (Additional file 1). On average across all cities and insecticides *A. aegypti* from urban populations had lower mortality to insecticides than those from suburban habitat (*P* < 0.01) (Fig. 3). Across all cities and environments bendiocarb caused significantly higher levels of mortality compared to alphacypermethrin (*P* < 0.01), deltamethrin (*P* < 0.01), and 0.75% permethrin (*P* < 0.01). However, these simple patterns had exceptions; in Yaoundé Bendiocarb had the lowest mortality rates and insecticide mortality was lower in suburban Garoua versus urban Garoua (Fig. 3).

Overall mortality to insecticides showed a strong latitudinal gradient, with higher mortality rates in northern cities (Fig. 3).

### Insecticide resistance profile for *Aedes albopictus*

**Fig. 4:**
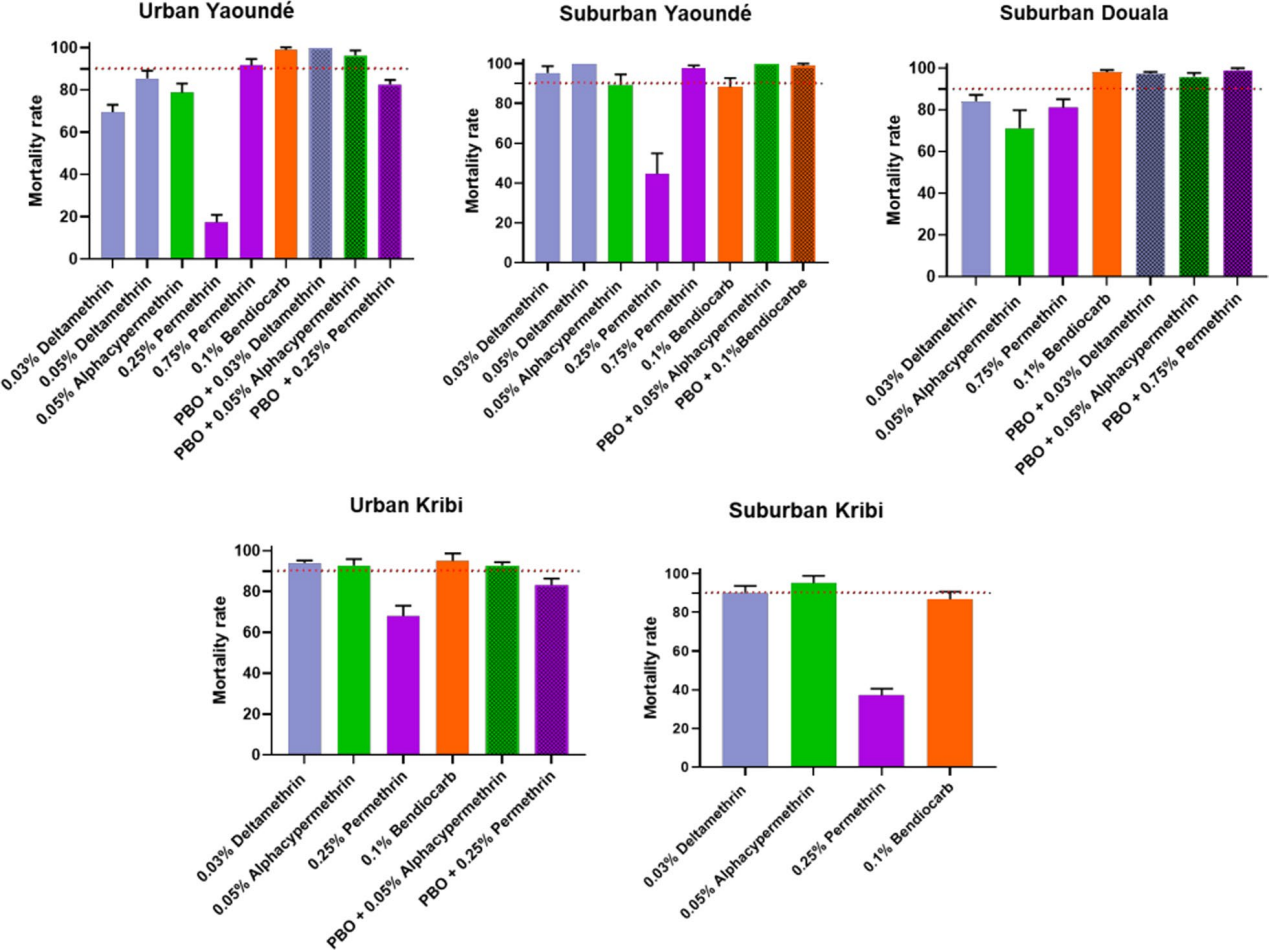
Mortality rates of adult *Aedes albopictus* from urban and suburban habitats in three cities in Cameroon 24 h after exposure to insecticides alone or with 1 h preexposure to synergist. Error bars represent standard error of the mean. *PBO* Piperonyl butoxide

Five *Ae. albopictus* populations were tested (urban Yaoundé, suburban Yaoundé, urban Kribi, suburban Douala, and suburban Kribi). The results are presented in Fig. 4. The analysis showed that resistance or probable resistance was observed to the three pyrethroids used. All populations tested with 0.25% permethrin showed high resistance with mortality rates between 17.35% (urban Yaoundé) and 81.15% (suburban Douala). When the dose of permethrin was increased to 0.75%, some recovery of susceptibility was restored. In urban Yaoundé, the 0.25% permethrin mortality rate was 17.35%, but when 0.75% permethrin was used the mortality rate increased to 91.59%. Two of five populations were found resistant to deltamethrin; urban Yaoundé with mortality rate of 69.47%, and suburban Douala with mortality rate of 84.11%, whereas the three others were shown to exhibit probable resistance (suburban Yaoundé, urban Kribi, and suburban Kribi). To alphacypermethrin, resistance was observed in suburban Douala with mortality rate of 71.21%, urban Yaoundé with mortality rate of 78.63%, and suburban Yaoundé with mortality rate of 89.40%; probable resistance was found in urban Kribi with mortality rate of 92.70%, and suburban Kribi with mortality rate of 95.17%. Moderate level of resistance was reported to bendiocarb with mortality rates varying from 86.75% in suburban Kribi to 98.95% in urban Yaoundé. Environment was not a statistically significant predictor for resistance in *Ae. albopictus.*

In cities where both *Ae. albopictus* and *Ae. aegypti* occur sympatrically (Douala, Kribi, and Yaoundé) *Ae. aegypti* had significantly higher levels of resistance than *Ae. albopictus*. The overall mean mortality to the insecticides standard doses tested were 54.7% and 80.1% for *Ae. aegypti* and for *Ae. albopictus* from sympatric cities (*P* < 0.02, *χ*^2^ = 6.61, df = 1).

### Tests with synergist PBO

A partial or full recovery of susceptibility to insecticides (permethrin, alphacypermethrin, deltamethrin and bendiocarb) was reported after PBO pre-exposure for both species (Figs. 2, 4).

For *Ae. albopictus* samples, a partial recovery of susceptibility was reported to permethrin from populations in urban Yaoundé (17.35% mortality without PBO and 82.54% after PBO exposure *P* < 0.001), suburban Douala (81.15% mortality without PBO and 99.1% after PBO exposure *P* > 0.05), and urban Kribi (68.08% mortality without PBO and 83.21% after PBO exposure *P* > 0.1). Susceptibility was partially recovered to alphacypermethrin in *Ae. albopictus* populations from urban Yaoundé (78.63% mortality without PBO and 96.36% after PBO exposure *P* > 0.05), suburban Douala (71.21% mortality without PBO and 95.94% after PBO exposure *P* > 0.01), and to bendiocarb in populations from suburban Yaoundé (88.18% mortality without PBO and 98.9% after PBO exposure *P* > 0.25). Full susceptibility recovery to deltamethrin was reported in urban Yaoundé (69.47% mortality without PBO and 100% after PBO exposure *P* < 0.005) and to alphacypermethrin in suburban Yaoundé 89.40% mortality without PBO and 100% after PBO exposure *P* > 0.25.

For *Ae. aegypti* sample*s*, a partial recovery of susceptibility was reported to permethrin in populations from urban Yaoundé (78.32% mortality without PBO and 92.18% after PBO exposure *P* > 0.1), suburban Douala (40.48% mortality without PBO and 62.13% after PBO exposure *P* < 0.01) and urban Ngaoundéré (85.89% mortality without PBO and 93.25% after PBO exposure *P* > 0.5). Also, susceptibility was partially recovered to alphacypermethrin in *Ae. aegypti* populations from urban Yaoundé (70.71% mortality without PBO and 95.45% after PBO exposure *P* > 0.01), suburban Douala (52.27% mortality without PBO and 54.27% after PBO exposure *P* > 0.75) and urban Ngaoundéré (88.52% mortality without PBO and 98.80% after PBO exposure *P* > 0.25), to deltamethrin in populations from urban Yaoundé (88.20% mortality without PBO and 98.03% after PBO exposure *P* > 0.25), suburban Douala (62.52% mortality without PBO and 68.83% after PBO exposure *P* > 0.25) and urban Ngaoundéré (83.77% mortality without PBO and 98.86% after PBO exposure *P* > 0.1), and to bendiocarb in populations from suburban Douala (91.61% mortality without PBO and 97.79% after PBO exposure *P* > 0.5).

### Gene expression

**Fig. 5:**
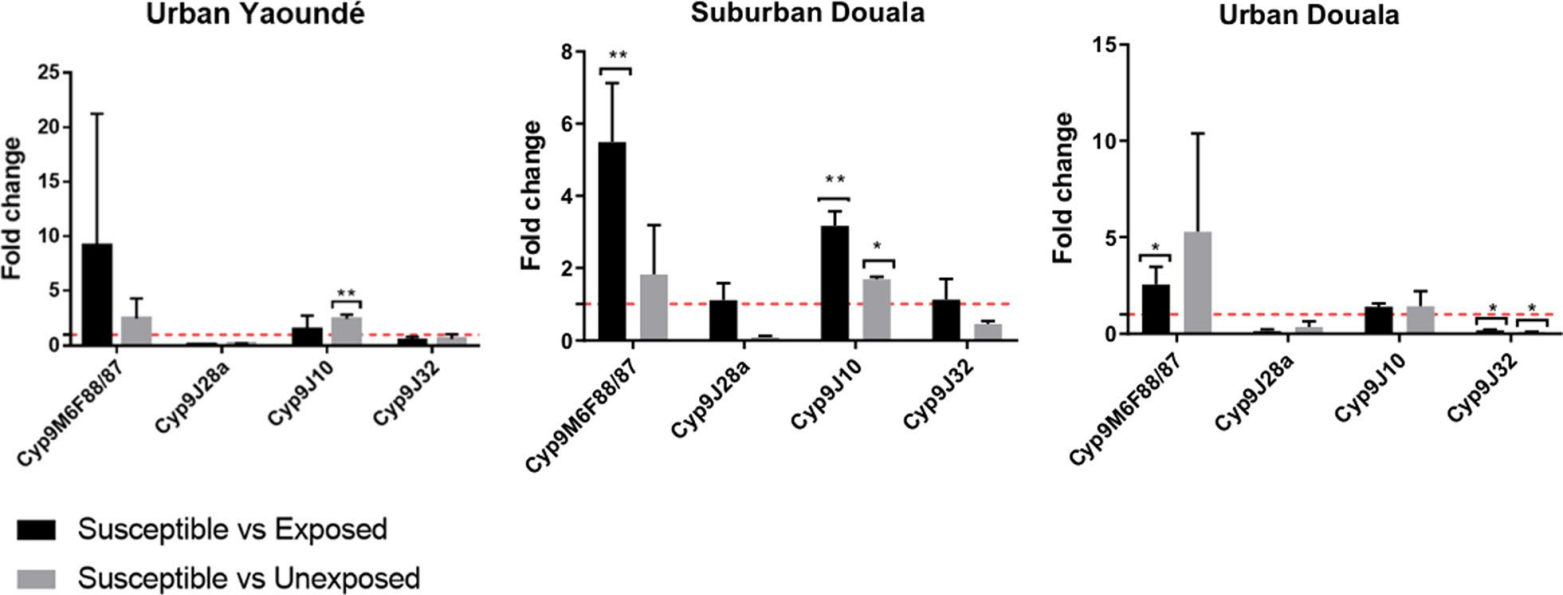
Expression of the *CYP9M6F88/87, CYP9J28a, CYP9J10, CYP9J32* genes in samples of *Aedes aegypti* from urban Yaoundé and urban and suburban Douala

Using qPCR, expression of four cytP450 genes were quantified in *Ae*. *aegypti* (*Cyp9M6F88/87*, *Cyp9J28a*, *Cyp9J10,* and *Cyp9J32*). Among them, two were significantly overexpressed in field populations compared to a susceptible lab strain (Fig. 5). *Cyp9M6F88/87* was overexpressed in urban Douala sample resistant to permethrin [(fold change (FC) = 2.54 ± 0.90, *P* = 0.016)] and suburban Douala samples resistant to deltamethrin (FC = 5.49 ± 1.64, *P* = 0.003); and *Cyp9J10* was overexpressed in suburban Douala (FC = 3.16 ± 0.40, *P* = 0.013).

**Fig. 6:**
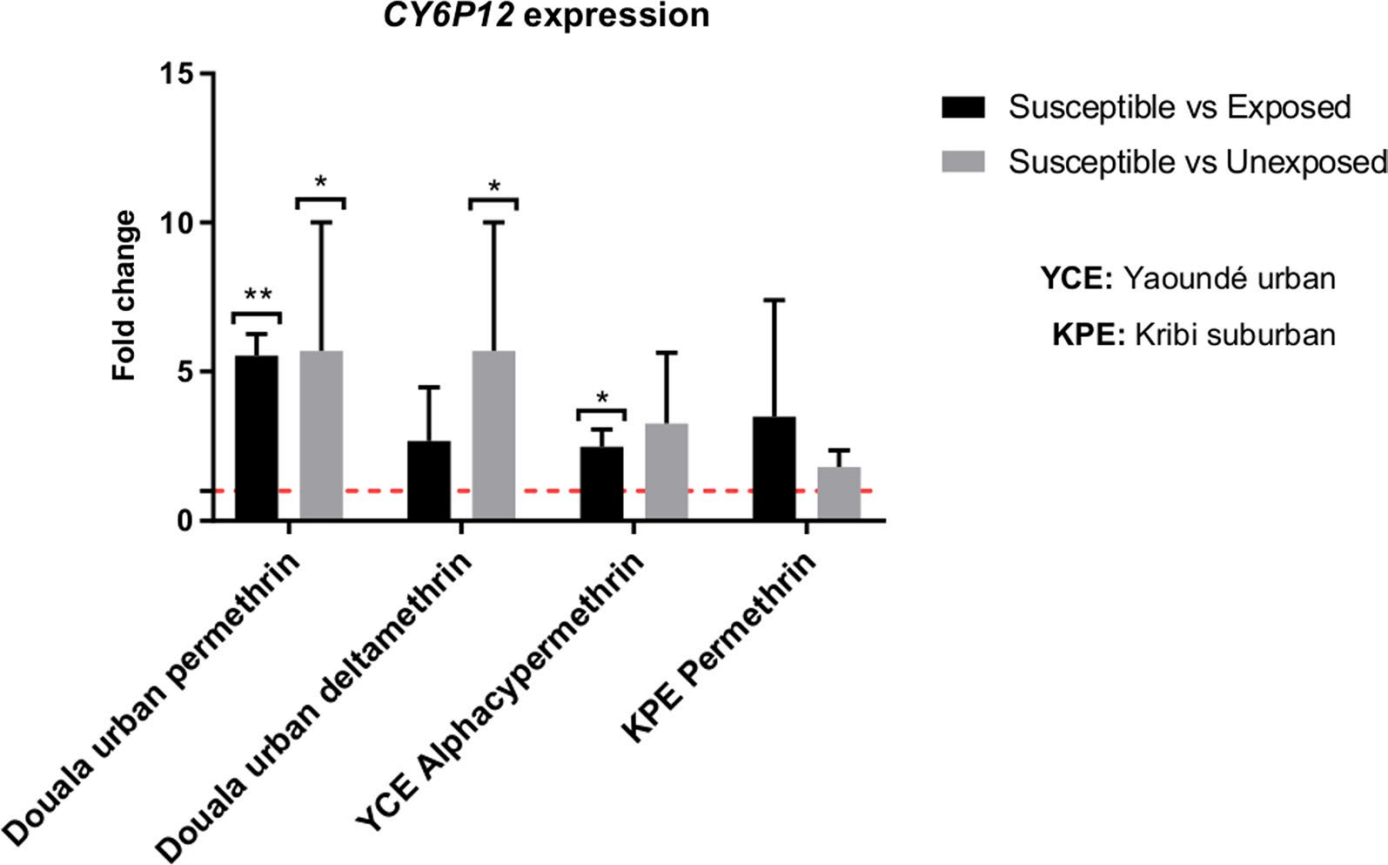
Expression of the *CYP6P12* gene in samples of *Aedes albopictus* from urban Yaoundé, suburban Kribi, and urban Douala

Only one gene (*Cyp6P12*) was assessed in *Ae*. *albopictus* populations. This gene was significantly more expressed in urban Douala samples resistant to permethrin, compared to a lab susceptible strain (FC = 5.54 ± 0.73, *P* = 0.001) and in urban Yaoundé samples resistant to alphacypermethrin (FC = 2.48 ± 0.57, *P* = 0.034). However, in urban Douala samples resistant to deltamethrin, *Cyp6P12* expression was not significantly different compared to susceptible strain (Fig. 6).

### Knockdown resistance (*kdr*) genotyping in* Ae. aegypti*

In this study, three *kdr* mutations were genotyped: V1016I, F1534C and V410L. The results are given in Tables 3, 4 and 5.

**Table 3:** 1016 genotype numbers and the allelic frequency of the Isoleucine (I) mutation of *Aedes aegypti*

Location	V1016 genotypes				Allelic frequencies	
	VV	VI	II	VV + VI + II	V	I
Urban Douala	21	6	0	27	0.89	0.1
Suburban Douala	22	8	0	30	0.87	0.13
Urban Yaoundé	27	2	1	30	0.93	0.07
Urban Maroua	13	14	2	29	0.69	0.31
Suburban Maroua	30	0	0	30	1	0
Urban Garoua	22	8	0	30	0.87	0.13
Suburban Garoua	27	2	1	30	0.93	0.07
Urban Ngaoundéré	21	8	1	30	0.83	0.17
Suburban Ngaoundéré	30	0	0	30	1	0
Urban Kribi	14	11	5	30	0.65	0.35
Total	227	59	10	296	0.87	0.13

V: Valine; I: isoleucine; VV: absence of the V1016I mutation; VI: presence of the V1016I mutation with 2 alleles: one resistant, allele I and another susceptible V allele; II: presence of the V1016I mutation with the 2 resistant alleles

**Table 4:** 1534 genotype numbers and the allelic frequency of the Cysteine (C) mutation of *Aedes aegypti*

Location	F1534 genotypes				Allelic frequencies	
	FF	FC	CC	FF + FC + CC	F	C
Urban Douala	0	3	26	29	0.05	0.95
Suburban Douala	1	5	24	30	0.12	0.88
Urban Yaoundé	27	2	1	30	0.93	0.07
Urban Maroua	8	1	19	28	0.30	0.70
Suburban Maroua	30	0	0	30	1	0
Urban Garoua	30	0	0	30	1	0
Suburban Garoua	26	2	1	29	0.93	0.07
Urban Ngaoundéré	18	5	7	30	0.68	0.32
Suburban Ngaoundéré	30	0	0	30	1	0
Urban Kribi	0	0	30	30	0	1
Total	170	18	108	296	0.60	0.40

F: phenylalanine; C: cysteine; F/F: absence of the F1534C mutation; F/C: presence of the F1534C mutation with 2 alleles: one resistant, allele C and another susceptible F allele; C/C: presence of the F1534C mutation with the 2 resistant alleles

**Table 5:** 410 genotype numbers and the allelic frequency of the Leucine (L) mutation of *Aedes aegypti*

Location	V410 genotypes				Allelic frequencies	
	VV	VL	LL	VV + VL + LL	V	L
Urban Douala	22	7	1	30	0.85	0.15
Suburban Douala	23	7	0	30	0.88	0.12
Urban Yaoundé	27	2	1	30	0.93	0.07
Urban Maroua	14	15	1	30	0.72	0.28
Suburban Maroua	30	0	0	30	1	0
Urban Garoua	22	8	0	30	0.87	0.13
Suburban Garoua	26	2	1	29	0.93	0.07
Urban Ngaoundéré	1	9	20	30	0.18	0.82
Suburban Ngaoundéré	30	0	0	30	1	0
Urban Kribi	17	9	4	30	0.72	0.28
Total	212	59	28	299	0.81	0.19

V: valine; L: leucine; VV: absence of the V410L mutation; VL: presence of the V410L mutation with 2 alleles: one resistant, allele L and another susceptible V allele; LL: presence of the V410L mutation with the 2 resistant alleles

For V1016I *kdr* genotyping, 296 samples were examined in total. Among them, 227 (76.69%) were susceptible (1016 V/V), 59 (19.93%) were heterozygote resistant (1016 V/I), and 10 (3.38%) were homozygote resistant (1016I/I). The allele frequencies were 86.66% and 13.34% for alleles V and I respectively.

**Fig. 7:**
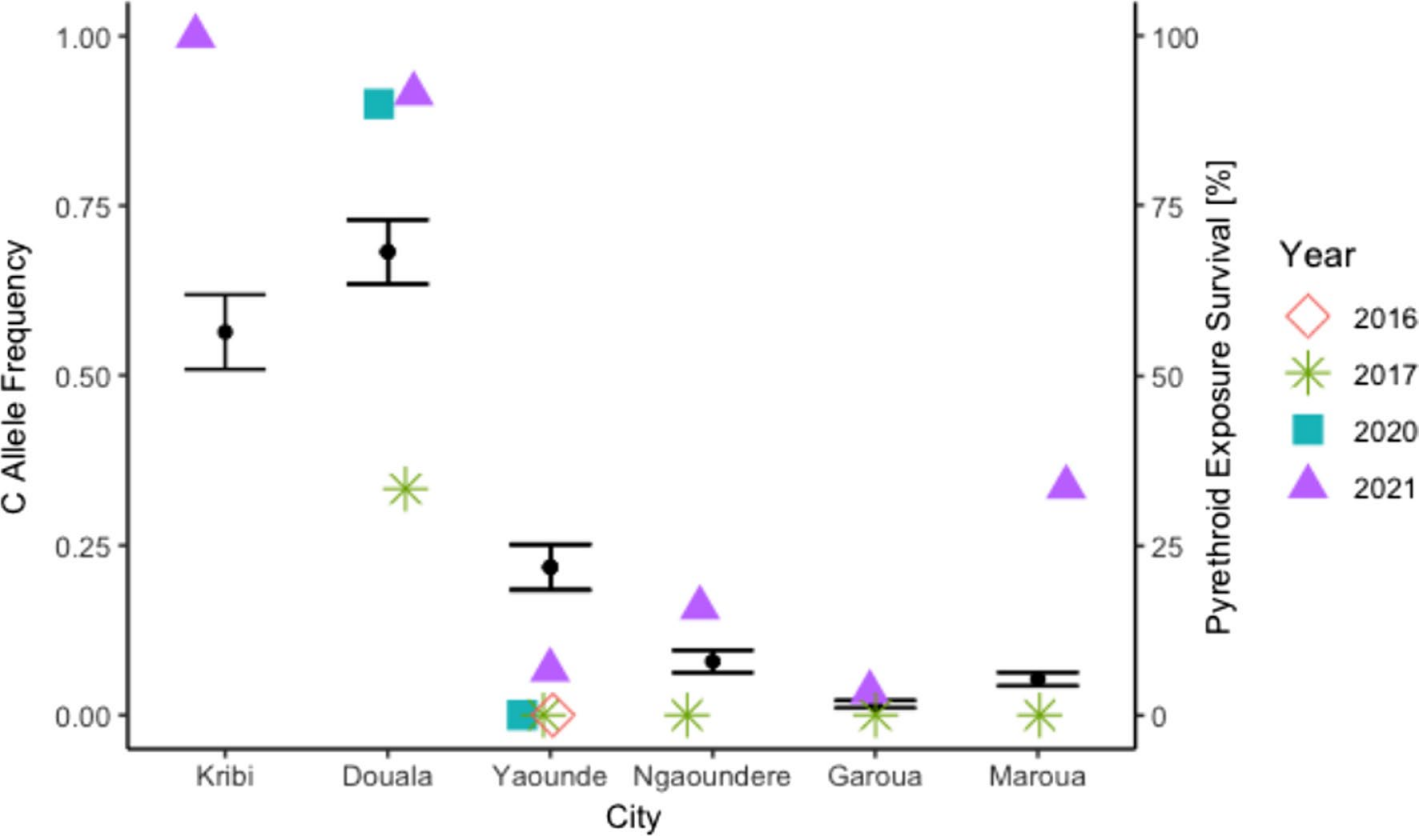
Frequency of the 1534C allele *kdr* mutation in *Aedes aegpyti* across study cities by year. Black circles represent mean pyrethroid survival (± 1 SE) from studied cities for 2021

We examined 296 samples for F1534C genotyping. Among them, 170 (57.43%) were susceptible (1534F/F), 18 (6.08%) were heterozygote resistant (1534F/C), and 108 (36.49.05%) were homozygote resistant (1534C/C). The allele frequencies were 60.47% and 39.53% for alleles F and C, respectively. C allele frequency had increased from levels first found in 2017 in 5 of the studied cities (Fig. 7).

We genotyped 299 samples for V410L *kdr* and 212 of samples (70.91%) were susceptible (410 V/V), 59 (19.73%) were heterozygote resistant (410 V/I), and 28 (9.36%) were homozygote resistant (410I/I). The allele frequencies were 80.77% and 19.23% for alleles V and L, respectively.

## Discussion

The aim of this study was to evaluate insecticides resistance profiles of *Ae*. *albopictus* and *Ae. aegypti* collected in several locations in Cameroon and explore the potential mechanisms. Analyses revealed resistance to all four insecticides tested (deltamethrin, alphacypermethrin, permethrin, and bendiocarb) for both *Aedes* species. While the loss of susceptibility to these insecticides in *Aedes* was previously reported in some locations in Cameroon [10, 28, 29] and outside Africa [30–33], this study expanded the spatial scale of insecticide resistance assessment in Cameroon while simultaneously comparing two species. The cause of resistance to insecticides in both *Ae. aegypti* and *Ae. albopictus* remains unclear as the use of insecticides against *Aedes* is scarce in African countries [10, 15, 30]. Nonetheless alleles, such as F1534C, that confer insecticide resistance continue to increase in frequency over time indicating selective pressure towards greater insecticide resistance. Given that the source of selective pressure for resistance is unknown, a return to susceptibility seems improbable [15] and could have operational consequences. The reduced susceptibility to the pyrethroids tested may pose a serious threat to future vector control programs, because pyrethroids are recommended for the control of adult *Aedes* mosquitoes, especially in case of disease outbreaks [34], and are commonly used in insecticide treated nets and uniforms.

*Aedes aegypti* populations from the northern part of Cameroon were more susceptible to pyrethroids than those from the southern part. This suggests that resistance to this insecticide family has not yet spread throughout the entire country and these insecticides are still effective in controlling *Aedes* in some locations of Cameroon. Previous studies on *Anopheles* in Cameroon showed the same latitudinal gradient to resistance distribution [35]. Climate is a potential contributing mechanism; while the relationship between insecticide resistance and climate for *Aedes* mosquitoes is largely unexplored, solar radiation and humidity were the highest-ranked predictor variable for resistance levels in Cameroonian *Anopheles* [35]. As with our study, *Anopheles* mosquitoes from wet tropical areas generally had higher levels of resistance than those from dry arid climates.

Pyrethroid resistance was more frequent in urban settings, which mirrors patterns in *Anopheles* mosquitoes in Cameroon [36]. This may be the result of a more intensive use of insecticide treated nets (ITN) and household insecticide (e.g., mosquito coils) usage in urban settings. However, both *Ae. aegypti* and *Ae. albopictus* feed primarily outdoors and during daylight in Cameroon and are thus unlikely to encounter ITNs [37, 38]. Land use could also affect resistance by determining the availability of larval habitat types since *Ae. aegypti* insecticide resistance levels can vary between larvae that developed in tires versus water containers [15].

With pre-exposure to PBO, partial or full recovery of susceptibility to all insecticides tested in *Ae. aegypti* and *Ae. albopictus* was observed. Similar results have been seen in several other African countries [10, 15, 28, 29, 33], as well as outside Africa [16, 17]. These findings suggested the important role played by P450 genes in the resistance to pyrethroids (deltamethrin, alphacypermethrin, and permethrin) and carbamates (bendiocarb). These observations were confirmed by the overexpression of genes such as *Cyp9M6F88/87* and *Cyp9J1*0 in some *Ae. aegypti* populations and *Cyp6P12* in *Ae. albopictus* populations in Cameroon. The implication of these genes in metabolic resistance were previously demonstrated [16–19, 39].

Three possible *kdr* mutations were genotyped in this study: F1534C, V1016I, and V410L, which are involved in pyrethroids resistance in *Aedes* mosquito [11, 40–42]. These three *kdr* mutations have been previously reported in Africa [15, 42–44], but only F1534C and V1016I were found previously in Cameroon [9, 10]. This study revealed for the first time the presence of a V410L mutation in *Ae. aegypti* in Cameroon. This mutation was first reported in 2017 in Brazil [7], and its first evidence in Africa was in 2020 in Angola [11]. V410L, alone confers low levels of resistance to insecticides, but when it co-occurs with V1016I or F1534C it yields higher levels of resistance [7, 11]. The frequency of V410L was moderate compared to frequencies in Angola, supporting the hypothesis of a novel introduction of this mutation. The F1534C and V1016I *kdr* mutations are common in *Ae*. *aegypti* and have a worldwide distribution [4]. V1016I and V410L have a moderate frequency, while F1534C has a high frequency and has increased in *Ae. aegypti* populations across Cameroon since the mutation was first observed there in 2017. This result supports the observation made previously in Cameroon suggesting an introduction and a gradual spread of the F1534C mutation in the country [10]. Broadly, the detection of *kdr* mutations are more frequent in Africa than was observed in the past [45]. Faced with their involvement in pyrethroid resistance, these three *kdr* mutations could have an impact on vector control measures.

The main limitation of the study is that we did not assess whether there is an association between the phenotypic resistance observed and the *kdr* mutations detected.

## Conclusions

Our study revealed the loss of susceptibility to four insecticides: deltamethrin, alphacypermethrin, permethrin, and bendiocarb, for both *Ae. aegypti* and *Ae. albopictus*. These insecticides are most likely to be used for mosquito control in outbreak responses and for reducing disease risk to deployed military personnel, and this study suggests these mitigation strategies will have limited efficacy. The level of loss of susceptibility varied according to the city, land use class, and species. Our results showed that several mechanisms are involved in resistance, and this can impact the strategies of *Aedes* control in Cameroon including the first detection of the V410L mutation in Cameroon. Further investigations including testing novel insecticides are needed to help to put in place effective strategies to control arbovirus vectors in Cameroon.

## Supplementary Information

**Additional file 1.** Logistic regression of *Aedes aegypti* mortality to insecticides across cities and habitat types*.* Suburban habitat, city of Douala, and Bendiocarb were reference levels.

## Data Availability

All the relevant data generated during this study are included in the manuscript.

## Abbreviations

DNA: Deoxyribonucleic acid
kdr: Knockdown resistance
RNA: Ribonucleic acid
PBO: Piperonyl butoxide
PCR: Polymerase chain reaction
*VGSC*: Voltage-gated sodium channel
WHO: World Health Organization
