# Supplementary material for: Spatial distribution of insecticide resistant populations of Aedes aegypti and Ae. albopictus and first detection of V410L mutation in Ae. aegypti from Cameroon
Source: Infect Dis Poverty. 2022 Aug 17;11:90. doi: 10.1186/s40249-022-01013-8 (PMC9382841; doi:10.1186/s40249-022-01013-8)
Supplement: Supplementary file 1 — Additional file 1. Logistic regression of Aedes aegypti mortality to insecticides across cities and habitat types. Suburban habitat, city of Douala, and Bendiocarb were reference levels. [file 40249_2022_1013_MOESM1_ESM.doc]

***Additional file 1: Logistic regression of Aedes aegypti mortality to insecticides across cities and habitat types. Suburban habitat, city of Douala, and Bendiocarb were reference levels.***

| **Predictor** | **Estimate** | **Std. Error** | **Z-value** | **Pr(>|z|)** |
| --- | --- | --- | --- | --- |
| (Intercept) | 2.42 | 0.37 | 6.56 | >0.001 |
| Habitat Urban | 0.49 | 0.59 | 0.84 | 0.404 |
| City Garoua | 0.47 | 0.59 | 0.80 | 0.425 |
| City Kribi | 0.08 | 0.69 | 0.12 | 0.904 |
| City Maroua | -0.22 | 0.51 | -0.44 | 0.661 |
| City Ngaoundéré | 0.06 | 0.54 | 0.12 | 0.905 |
| City Yaoundé | -2.11 | 0.51 | -4.14 | >0.001 |
| Insecticide Alphacypermethrin 0.05 | -2.32 | 0.42 | -5.55 | >0.001 |
| Insecticide Deltamethrin 0.03 | -1.93 | 0.42 | -4.57 | >0.001 |
| Insecticide Permethrin 0.25 | 1.38 | 0.82 | 1.69 | 0.092 |
| Insecticide Permethrin 0.75 | -2.81 | 0.42 | -6.61 | >0.001 |
| Habitat Urban: City Garoua | -1.20 | 0.82 | -1.46 | 0.144 |
| Habitat Urban: City Kribi | NA | NA | NA | NA |
| Habitat Urban: City Maroua | 1.79 | 1.22 | 1.47 | 0.142 |
| Habitat Urban: City Ngaoundéré | 0.04 | 0.87 | 0.05 | 0.960 |
| Habitat urban: City Yaoundé | NA | NA | NA | NA |
| Habitat Urban: Insecticide Alphacypermethrin 0.05 | -3.27 | 0.75 | -4.36 | >0.001 |
| Habitat Urban: Insecticide Deltamethrin 0.03 | -2.21 | 0.67 | -3.29 | 0.001 |
| Habitat Urban: Insecticide Permethrin 0.25 | -2.59 | 1.01 | -2.58 | 0.010 |
| Habitat Urban: Insecticide Permethrin 0.75 | -2.95 | 0.78 | -3.80 | >0.001 |
| City Garoua: Insecticide Alphacypermethrin 0.05 | 20.31 | 2163.56 | 0.01 | 0.993 |
| City Kribi: Insecticide Alphacypermethrin 0.05 | 2.80 | 0.83 | 3.37 | 0.001 |
| City Maroua: Insecticide Alphacypermethrin 0.05 | 3.06 | 0.71 | 4.29 | >0.001 |
| City Ngaoundéré: Insecticide Alphacypermethrin 0.05 | 3.23 | 0.82 | 3.93 | >0.001 |
| City Yaoundé: Insecticide Alphacypermethrin 0.05 | 5.69 | 0.70 | 8.09 | >0.001 |
| City Garoua: Insecticide Deltamethrin 0.03 | 19.92 | 2163.56 | 0.01 | 0.993 |
| City Kribi: Insecticide Deltamethrin 0.03 | 1.18 | 0.76 | 1.55 | 0.121 |
| City Maroua: Insecticide Deltamethrin 0.03 | 4.17 | 1.15 | 3.64 | >0.001 |
| City Ngaoundéré: Insecticide Deltamethrin 0.03 | 20.33 | 2165.31 | 0.01 | 0.993 |
| City Yaoundé: Insecticide Deltamethrin 0.03 | NA | NA | NA | NA |
| City Garoua: Insecticide Permethrin 0.25 | 0.18 | 1.37 | 0.13 | 0.898 |
| City Kribi: Insecticide Permethrin 0.25 | -2.92 | 0.82 | -3.58 | >0.001 |
| City Maroua: Insecticide Permethrin 0.25 | 0.24 | 1.14 | 0.22 | 0.830 |
| City Ngaoundéré: Insecticide Permethrin 0.25 | NA | NA | NA | NA |
| City Yaoundé: Insecticide Permethrin 0.25 | NA | NA | NA | NA |
| City Garoua: Insecticide Permethrin 0.75 | NA | NA | NA | NA |
| City Kribi: Insecticide Permethrin 0.75 | NA | NA | NA | NA |
| City Maroua: Insecticide Permethrin 0.75 | NA | NA | NA | NA |
| City Ngaoundéré: Insecticide Permethrin 0.75 | NA | NA | NA | NA |
| City Yaoundé: Insecticide Permethrin 0.75 | 6.24 | 0.73 | 8.51 | >0.001 |
| Habitat Urban: City Garoua: Insecticide Alphacypermethrin 0.05 | 14.00 | 2163.56 | -0.01 | 0.995 |
| Habitat Urban: City Kribi: Insecticide Alphacypermethrin0.05 | NA | NA | NA | NA |
| Habitat Urban: City Maroua: Insecticide Alphacypermethrin 0.05 | -0.12 | 1.41 | -0.09 | 0.931 |
| Habitat Urban: City Ngaoundéré: Insecticide Alphacypermethrin 0.05 | 1.40 | 1.20 | 1.17 | 0.242 |
| Habitat Urban: City Yaoundé: Insecticide Alphacypermethrin 0.05 | NA | NA | NA | NA |
| Habitat Urban: City Garoua: Insecticide Deltamethrin 0.03 | 13.41 | 2163.56 | -0.01 | 0.995 |
| Habitat Urban: City Kribi: Insecticide Deltamethrin 0.03 | NA | NA | NA | NA |
| Habitat Urban: City Maroua: Insecticide Deltamethrin 0.03 | -1.46 | 1.69 | -0.86 | 0.388 |
| Habitat Urban: City Ngaoundéré: Insecticide Deltamethrin 0.03 | 17.56 | 2165.31 | -0.01 | 0.994 |
| Habitat Urban: City Yaoundé: Insecticide Deltamethrin 0.03 | NA | NA | NA | NA |
| Habitat Urban: City Garoua: Insecticide Permethrin 0.25 | 2.68 | 1.69 | 1.59 | 0.112 |
| Habitat Urban: City Kribi: Insecticide Permethrin 0.25 | NA | NA | NA | NA |
| Habitat Urban: City Maroua: Insecticide Permethrin 0.25 | -0.46 | 1.71 | -0.27 | 0.787 |
| Habitat Urban: City Ngaoundéré: Insecticide Permethrin 0.25 | NA | NA | NA | NA |
| Habitat Urban: City Yaoundé: Insecticide Permethrin 0.25 | NA | NA | NA | NA |
| Habitat Urban: City Garoua: Insecticide Permethrin 0.75 | NA | NA | NA | NA |
| Habitat Urban: City Kribi: Insecticide Permethrin 0.75 | NA | NA | NA | NA |
| Habitat Urban: City Maroua: Insecticide Permethrin 0.75 | NA | NA | NA | NA |
| Habitat Urban: City Ngaoundéré: Insecticide Permethrin 0.75 | NA | NA | NA | NA |
| Habitat Urban: City Yaoundé: Insecticide Permethrin0.75 | NA | NA | NA | NA |
